# Multiparameter Analysis Using ^18^F-FDG PET/CT in the Differential Diagnosis of Pancreatic Cystic Neoplasms

**DOI:** 10.1155/2021/6658644

**Published:** 2021-04-07

**Authors:** Guanyun Wang, Haodan Dang, Peng Yu, Honghong Liu, Yue Wu, Shulin Yao, Jiahe Tian, Huiyi Ye, Baixuan Xu

**Affiliations:** ^1^Department of Nuclear Medicine, The First Medical Centre, Chinese PLA General Hospital, Fuxing Road 28, Beijing 100853, China; ^2^Medical School of Chinese PLA, Fuxing Road 28, Beijing 100853, China; ^3^Diagnostic Imaging, Siemens Healthineers Ltd., Beijing 100102, China; ^4^Department of Radiology, The First Medical Centre, Chinese PLA General Hospital, Fuxing Road 28, Beijing 100853, China

## Abstract

**Purpose:**

To evaluate multiparametric analysis in differential diagnosis between pancreatic serous cystic neoplasms (SCNs) and mucinous cystic neoplasms (MCNs) as well as the differentiation of the benign and malignant MCNs with ^18^F-FDG (18-fluorodeoxyglucose) PET/CT (positron emission tomography).

**Methods:**

Forty patients with total of 41 lesions (SCNs: 27/41; MCNs: 14/41), who were preoperatively examined with ^18^F-FDG PET/CT, were retrospectively analyzed. Multiple quantitative parameters using conventional and texture features were included. The combined model was established with complementary PET/MR parameters. The differential diagnostic efficacy of each independent parameter and the combined model were evaluated with receiver operating characteristic (ROC) analysis. Integrated discriminatory improvement (IDI) and net reclassification improvement (NRI) were used to evaluate improvement of diagnostic efficacy by using combination of multiple parameters.

**Results:**

Among all independent parameters, the percentile 5th (0.88 ± 0.38 vs 0.47 ± 0.23, *P* < 0.001) showed the highest discriminative diagnostic value. The combination of multiple parameters can improve the differential diagnostic efficacy of SCNs and MCNs (sensitivity = 71.4%, specificity = 77.8%, and AUC = 0.788), and the addition of texture parameters to the conventional parameters allowed a significant reclassification with IDI = 0.236 (95% CI: 0.095–0.377) and categorical NRI = 0.434 (95% CI: 0.030–0.838). SURmax (tumor to normal pancreas ratio, T/P) and SURmax (tumor to aorta ratio, T/A) both showed the highest discriminative diagnostic value (sensitivity = 100.0%, specificity = 70.0%, AUC = 0.900, and Youden index = 0.700) in the differential diagnosis of benign and malignant MCNs, with the cutoff values of 0.84 and 0.90, respectively.

**Conclusion:**

Combination of multiple parameters using ^18^F-FDG PET/CT could further improve differentiation between pancreatic SCNs and MCNs. SURmax (T/P) and SURmax (T/A) could improve differential diagnosis of benign and malignant MCNs.

## 1. Introduction

Serous cystic neoplasms (SCNs) and mucinous cystic neoplasms (MCNs) are two frequent cystic lesions of the pancreas. SCNs are considered as a benign lesion which predominantly occurs at the tail of the pancreas among older women, and the chance of malignant transformation is less than 1% [1, 2]. MCNs often occur in middle-aged women located in the body or tail of the pancreas, and the malignant transformation risk ranges from 10% to 17% [3, 4]. Therefore, distinguishing between the two tumors by noninvasive means has a particular clinical value.

Recently, the routine imaging examinations for pancreatic cystic neoplasms were performed with ultrasound, computed tomography (CT), and MR imaging (MRI) [3]. Imaging modalities like multidetector CT (MDCT), MR cholangiopancreatography (MRCP), and endoscopic ultrasound (EUS) have provided diagnostic value in identifying pancreatic cystic lesions. Furthermore, positron emission tomography/computed tomography (PET/CT) yields biological information which is beneficial for surgical resection and thus significantly improves the clinical management [5].

Multiparameter analysis is widely used in diagnostic imaging, especially for radiomics which is defined as high-throughput extraction of a large number of features from medical images [6, 7]. The predictive models based on multiparameter analysis have been developed in many studies for various clinical tasks such as preoperative diagnosis, evaluation of treatment response, and prognosis, compared to the conventional independent parameters [8–10]. Multiple imaging features based on histogram, texture, and radiomics analysis can be used to quantitatively evaluate the intertumoral heterogeneity on ^18^F-FDG PET/CT [11].

To the best of our knowledge, no published study has analyzed the histogram and texture features of ^18^F-FDG PET/CT to differentiate between SCNs and MCNs. The present study based on conventional and texture analysis with ^18^F-FDG PET/CT investigated the diagnostic efficacy in the differential diagnosis of pancreatic SCNs and MCNs by using multiple parameters independently and in combination and evaluated the ^18^F-FDG PET/CT in the differential diagnosis between benign and malignant MCNs.

## 2. Patients and Methods

### 2.1. Patients

This study was approved by the institutional ethics committee of the General Hospital of the People's Liberation Army. From May 2012 to January 2020, 40 patients with the suspicion of cystic pancreatic neoplasms were included in this retrospective study. The inclusion criteria comprised the following: [1] clinical diagnosis of cystic pancreatic neoplasms by conventional imaging (37 patients with ultrasound; 1 patient with CT; 2 patients with MR); [2] performed with ^18^F-FDG PET/CT before treatment; [3] pathological diagnosis of SCNs or MCNs; and benign or malignant lesions based on histological findings or liquid-based cytology. Among all included patients, 41 lesions were analyzed, including 27 SCNs (65.9%) and 14 MCNs (34.1%). In the 14 MCNs lesions, 4 were malignant (28.6%) and 10 were benign (71.4%).

### 2.2. PET/CT Scanning and Image Interpretation

All patients were scanned with ^18^F-FDG PET/CT (Biograph 64, Siemens, Germany). Patients fasted for six hours with plasma glucose levels under 11.1 mmol/L and rested for at least 20 min in a quiet waiting room before intravenous administration of 3.70–4.44 MBq/kg (0.10–0.12 mCi/kg) ^18^F-FDG (^18^F-FDG; Atomic High-Tech Co., Ltd., radiochemical purity of >95%). PET/CT scan was performed 50 minutes after injection, beginning from the skull base to the upper femur in free-breathing mode. The low-dose CT (LDCT) parameters were voltage = 120 kV, current = 100 mAs, rotation = 0.8, layer thickness = 5 mm, and pitch = 1. PET acquisition parameters were 3-dimensional mode, 2 min/bed (30% overlap), and 4-5 beds/person. The PET images were reconstructed with CT attenuation correction (AC) by using the ordered subset expectation maximization algorithm (OSEM) with 3 iterations, 21 subsets, and Gaussian filter half-height width = 4.0 mm.

Visual analysis on ^18^F-FDG PET/CT images was performed to differentiate malignant and benign lesions. Lesions were indicated as malignant with one or all of the following findings: (1) Lesions were found with increased uptake of FDG on PET images; (2) Lesions showed morphological changes of the pancreatic parenchyma, unclear margin of the tumor, dilatation of the pancreatic duct, vascular invasion, and enlarged lymph nodes on CT images.

### 2.3. Image Analysis

Multiparametric Analysis prototype (Siemens, Germany), a dedicated prototype postprocessing tool, was used for imaging analysis. Quantitative analyses were performed by two experienced nuclear medicine physicians (WGY and DHD). If the results were inconsistent between the two physicians, the process would be repeated two weeks later and reached a consensus. A two-dimensional region of interest (ROI) was delineated manually around the tumoral lesion on each layer of transaxial CT images to form a three-dimensional volume of interest (VOI). The texture features included standard deviation (SD), median, percentiles (5th, 95th), skewness, kurtosis, diffEntropy, diffVariance, contrast, and entropy by VOI-based signal intensity histogram analysis. In addition, the conventional PET/CT metabolic parameters including SUVmax (maximum standardized uptake value), SUVmean (mean standard uptake value), MTV (metabolic tumor volume), and TLG (total lesion glycolysis, SUVmean × MTV) were collected. Advanced PET/CT metabolic parameters including SURmax (T/P) (SUR: standardized uptake ratio; T/P: tumor/normal pancreas), SURmean (T/P), MTV (T/P), TLG (T/P), SURmax (T/A) (tumor/aorta), SURmean (T/A), MTV (T/A), and TLG (T/A) were also calculated.

### 2.4. Reference Standard

For pathology diagnosis, SCNs are the cyst-forming serous epithelium composed of cuboidal cells with clear glycogen-rich cytoplasm and bland cytology [12]. MCNs include an epithelium lined by tall, columnar, and mucin-producing cells and a subepithelial ovarian-type stroma [13]. Malignancy is identified based on invasion of the pancreatic parenchyma or metastases [14].

### 2.5. Statistical Analysis

All data were analyzed by using the R software (version 4.0.2; Bell Laboratories, USA). Continuous variables were presented as mean ± SD, and categorical variables were presented as percentages. The distribution of baseline characteristics and PET/CT multiple parameters among the two groups (SCNs and MCNs) were analyzed using the Student *t*-test (Levene's test was used to adjust the variances) and chi-square test. The optimal cutoff values of baseline characteristics and PET/CT parameters for the differentiation between two groups were determined by the highest Youden's index using receiver operating characteristic (ROC) analyses. Logistic regression was used to select the efficient parameters for the combined model. The integrated discriminatory improvement (IDI) and net reclassification improvement (NRI) were calculated for comparison of diagnostic models with or without texture parameters of PET/CT. *P* < 0.05 was considered as a statistically significant difference.

## 3. Results

**Figure 1:**
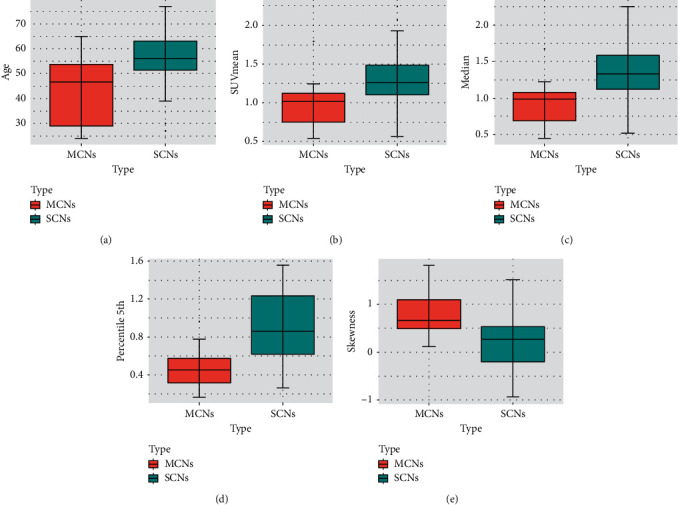
Distribution of the age (a), SUVmean (b), median (c), percentile 5th (d), and skewness (e) of serous cystic neoplasm (SCN, blue) and mucinous cystic neoplasm (MCN, red) patients. The age, SUVmean, median, and percentile 5th of the SCN group were higher than those of the MCN group (56.67 ± 12.24 vs 43.50 ± 13.27, *P*=0.003; 1.33 ± 0.43 vs 0.99 ± 0.33, *P*=0.014; 1.33 ± 0.14 vs 0.94 ± 0.33, *P*=0.006; 0.88 ± 0.38 vs 0.47 ± 0.23, *P* < 0.001). The skewness of the SCN group was lower than that of the MCN group (0.78 ± 0.45 vs 0.21 ± 0.57, *P*=0.002).

Table 1 summarizes the baseline characteristics and PET/CT parameters between the SCN and MCN lesions. The results showed that age (56.67 ± 12.24 vs 43.50 ± 13.27, *P*=0.003), SUVmean (1.33 ± 0.43 vs 0.99 ± 0.33, *P*=0.014), median (1.33 ± 0.14 vs 0.94 ± 0.33, *P*=0.006), percentile 5th (0.88 ± 0.38 vs 0.47 ± 0.23, *P* < 0.001), and skewness (0.21 ± 0.57 vs 0.78 ± 0.45, *P*=0.002) were statistically different between the two groups (SCNs vs MCNs) (Figure 1). The ROC analysis indicated that the percentile 5th showed the highest discriminative diagnostic value with a cutoff value of 0.78 (sensitivity = 92.9%, specificity = 63.0%, AUC = 0.780, and Youden index = 0.558). The model based on the logistic regression with only conventional and advanced metabolic parameters of PET resulted in an AUC of 0.788 (95% CI: 0.640–0.915), while the combined model with multiple metabolic parameters and texture parameters of PET/CT resulted in an AUC of 0.810 (95% CI: 0.661–0.937). The model is shown below.

(1) $$\begin{array}{c} y=\frac{1}{1+e^{-\left(-4.75\;\times \text{ median }+4.54\;\times \text{ SUVmean}-1.17\;\times \text{ percentile }5\text{th }+\;0.31\;\times \text{ skewness}-1.09\;\right)}}. \end{array}$$

**Figure 2:**
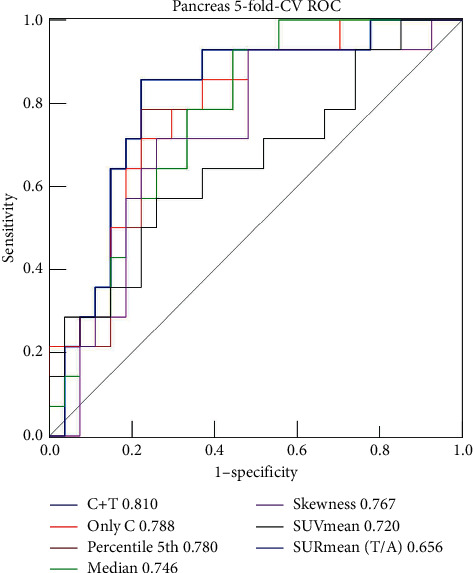
The ROC curves of baseline characteristics and top 5 independent metabolic parameters and the combined model.

**Figure 3:**
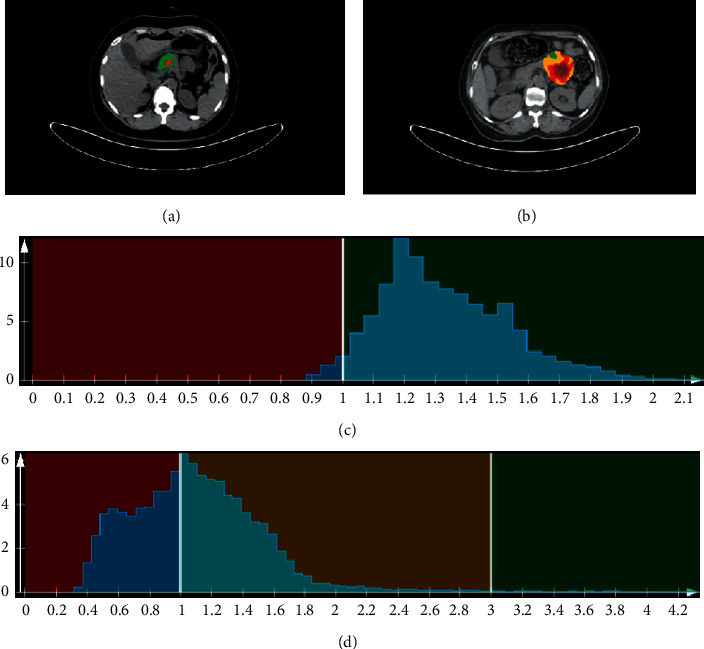
(a) PET/CT image of a 30-year-old female patient. The pathological result was SCNs. (b) PET/CT image of a 57-year-old female patient. The pathological result was MCNs. (c) and (d) are the VOI-based texture analysis histograms of PET image in (a) and (b), respectively. The histogram analysis showed skewness of each VOI as 0.612 and 1.811 in (c) and (d), respectively, and kurtosis as 0.168 and 6.979 in (c) and (d), respectively.

The diagnostic efficiencies of the top five independent parameters and the combined models are shown in Figure 2 and Table 2. All accuracy analyses were based on cross-validation. Finally, according to the analyses of IDI and NRI in Table 3, the addition of texture parameters to the conventional parameters allowed a significant reclassification with IDI = 0.236 (95% CI: 0.095–0.377) and categorical NRI = 0.434 (95% CI: 0.030–0.838), which showed the benefits of statistical diagnostic with multiparametric combination in differential diagnosis of the cystic pancreatic neoplasms. PET/CT images and the relative texture analysis histograms of SCNs and MCNs are shown in Figure 3.

In all 14 MCNs lesions, 4 were malignant according to pathological results. For visual analysis, the diagnostic sensitivity, specificity, and accuracy were 75.0%, 90.0%, and 78.6%, respectively. For quantitative analysis, three of the conventional PET/CT metabolic parameters showed significant differences between benign and malignant groups: TLG (malignant group: 140.35 ± 106.41, benign group: 33.48 ± 26.79, *P*=0.009), SURmax (T/P) (malignant group: 2.44 ± 1.43, benign group: 1.01 ± 0.61, *P*=0.018), and SURmax (T/A) (malignant group: 1.71 ± 1.05, benign group: 0.66 ± 0.42, *P*=0.024). The ROC analysis indicated that SURmax (T/P) and SURmax (T/A) at cutoff values of 0.84 and 0.90, respectively, showed highest discriminative diagnostic value (sensitivity = 100.0%, specificity = 70.0%, AUC = 0.900, and Youden index = 0.700).

## 4. Discussion

At present, there were few studies about PET/CT in the diagnosis of pancreatic cystic tumors. Among all ^18^F-FDG PET/CT metabolic parameters, our current findings indicated that the percentile 5th with texture analysis was the best to distinguish between SCNs and MCNs independently. Percentile 5th represents the 5th percentile of the set of grey levels of the voxels included in the VOI segmented as PET positive lesions, which is an intensity-based statistical feature. The PET histogram of MCNs displays a relatively steep peak with some wider slopes, and this may show more solid components in MCNs (Figure 3). Thus, higher skewness and kurtosis were detected in the MCN group, which indicated a higher heterogeneity. Our result suggested that the combination of conventional and texture metabolic parameters of ^18^F-FDG PET/CT significantly improved differentiation between SCNs and MCNs. Furthermore, we found that PET/CT parameters, including SURmax (T/P) and SURmax (T/A), were both significantly higher in malignant group than in benign group of MCNs. And these parameters were found to be more effective than visual analysis in differentiating between benign and malignant MCNs, which would benefit the treatment planning and prognosis for patients.

SCNs are considered as benign lesions, and regular clinical evaluation is suggested for clinical follow-up of these lesions. When the lesions are larger than 4 cm or have a macrocystic appearance, surgery should be the first choice because they are more likely to be symptomatic or malignant transformation [15, 16]. MCNs are mainly found in large size, septal, thick-walled cysts and are filled with mucus and occasionally calcification [4]. Compared to SCNs, MCNs were found with high potential of malignant transformation. Hence, all suspected MCN lesions were recommended to be surgically removed with histological confirmation obtained for further treatment management [17, 18]. Currently, differentiation between SCN and MCN using CT or MR imaging is very challenging. Many asymptomatic or accidental cases have similar imaging findings on CT or MRI [17, 19]. Mohamed et al. previously reported that the diagnostic accuracy of CT in cystic pancreatic lesions was low with precision between 39% and 61.4% [20]. MRI has higher diagnostic efficiency, as it has a higher morphological resolution for evaluation of multiplicity of cystic lesions and main pancreatic ductal communication [5, 21]. In recent studies, diagnostic accuracy of MRI varied between 50% and 86% and was able to identify aggressive behavior of malignant tumors [20, 22, 23]. However, main drawbacks of MRI included multiple artifacts, limited evaluation of calcifications, and long scanning time which make it intolerable for patients with abdominal discomfort [20, 24]. Another modality is EUS, specifically EUS-fine-needle aspiration, which improves diagnostic accuracy by analyzing the cystic fluid [25]. The significant drawbacks of EUS are that it is an invasive operation, and sampling is difficult for lesions located in the body or tail of the pancreas. Thus, it is particularly important to distinguish the pathological features of the lesions using advanced imaging methods.

In most studies, evaluation of cystic pancreatic neoplasms using PET/CT has shown better sensitivity and diagnostic accuracy compared to CT [26, 27]. PET/CT outperforms CT in tumor staging, treatment guiding, and prognosis. In the present study, percentile 5th was the best parameter of texture analysis in distinguishing the SCNs and MCN with the AUC of 0.780. Grey levels in PET represent the uptake intensity [28], and MCNs were found with significantly lower values of percentile 5th than SCNs. The pathological feature of MCNs with large size, septal, thick-walled cysts filled with mucus may lead to the lower tracer uptake. The underlying mechanism still needs further investigation. The combination of the conventional and texture metabolic parameters increased the AUC value to 0.810. Texture analysis with various parameters reflecting distribution characteristics of voxel grey level could reveal the heterogeneity within tumors, while conventional metabolic parameters of PET/CT could not. Our results showed a significant difference in heterogeneity between SCNs and MCNs using texture analysis. The main reason was that SCNs are mainly composed of multiple microcapsules, presenting a honeycomb appearance and central scar [29]. Conversely, MCNs are mostly multilocular with separation and combined with calcification [5].

The key limitation of the study is small sample size, which could bias the statistics and diagnostic model. Secondly, most ^18^F-FDG PET/CT examinations were performed when the patient was suspected of malignant transformation, which could also lead to bias in the analysis. Thirdly, at least five morphologic subtypes of SCNs are described [30], but limited by the pathological results, we could not identify the morphologic subtypes of SCNs, like microcystic and macrocystic/oligocystic variants. This will affect the result of texture analysis to a certain extent. Large-scale and prospective multicenter study should be performed to further evaluate this subject in future, and the pathological results should be more detailed classification of morphologic subtypes.

In conclusion, multiple metabolic parameters of ^18^F-FDG PET/CT showed varying diagnostic efficacy in differentiation of SCNs and MCNs. The combination of conventional and texture parameters could further improve the accuracy of the differential diagnosis. PET/CT parameters could be more effective in differential diagnosis of MCNs between benign and malignant.

## Figures and Tables

**Table 1:** The differences in baseline characteristics and PET/CT parameters between serous cystic neoplasm (SCN) and mucinous cystic neoplasm (MCN) patients.

	Serous cystic neoplasms (*n* = 27)	Mucinous cystic neoplasms (*n* = 14)	*P*
Baseline characteristics			
Age (years)	56.67 ± 12.24	43.50 ± 13.27	
Female sex, *n* (%)	20 (74.07%)	12 (85.71%)	
Diameter (cm)	3.97 ± 1.88	4.34 ± 1.87	

Conventional metabolic parameters of PET/CT			
SUVmax	1.82 ± 0.50	1.75 ± 0.86	**0.763**
SUVmean	1.33 ± 0.43	0.99 ± 0.33	**0.014**
MTV	49.03 ± 59.59	58.50 ± 49.54	**0.613**
TLG	61.52 ± 79.29	64.01 ± 74.97	**0.923**

Advanced metabolic parameters of PET/CT			
SURmax (T/P)	1.43 ± 0.80	1.42 ± 1.09	**0.980**
SURmean (T/P)	1.13 ± 0.48	0.99 ± 0.67	**0.449**
MTV (T/P)	14.23 ± 19.84	13.79 ± 17.51	**0.945**
TLG (T/P)	19.22 ± 30.53	21.31 ± 40.67	**0.854**
SURmax (T/A)	1.15 ± 0.57	1.39 ± 1.23	**0.596**
SURmean (T/A)	0.92 ± 0.33	0.96 ± 0.79	**0.214**
MTV (T/A)	18.22 ± 27.46	13.21 ± 14.02	**0.527**
TLG (T/A)	20.54 ± 37.20	18.19 ± 30.83	**0.841**

Texture parameters of PET/CT			
SD	0.29 ± 0.12	0.40 ± 0.29	**0.194**
Median	1.33 ± 0.45	0.94 ± 0.33	**0.006**
Percentile 5th	0.88 ± 0.38	0.47 ± 0.23	＜**0.001**
Percentile 95th	1.82 ± 0.50	1.75 ± 0.86	**0.763**
Skewness	0.21 ± 0.57	0.78 ± 0.45	**0.002**
Kurtosis	0.01 ± 0.85	0.69 ± 1.96	**0.124**
Diffentropy	0.26 ± 0.16	0.26 ± 0.16	**0.903**
Diffvariance	0.04 ± 0.04	0.05 ± 0.04	**0.801**
Contrast	0.07 ± 0.06	0.08 ± 0.07	**0.507**
Entropy	0.36 ± 0.30	0.42 ± 0.33	**0.549**

SUV : standardized uptake value; MTV : metabolic tissue volume; TLG : total lesion glycolysis; SUR: standardized uptake ratio; T/P : tumor/normal pancreas; T/A : tumor/aorta; SD : standard deviation.

**Table 2:** Differential diagnostic efficiency of top 5 independent metabolic parameters in baseline characteristics between the SCN and MCN groups with receiver operating characteristic analysis.

Variable	AUC	Sensitivity (%)	Specificity (%)	Youden index	*P*
SUVmean	0.720	71.4	74.1	0.588	**0.011**
SURmean (T/A)	0.656	57.1	74.1	0.312	**0.100**
Median	0.746	78.6	77.8	0.563	**0.003**
Percentile 5th	0.780	92.9	63.0	0.558	**＜0.001**
Skewness	0.767	92.9	55.6	0.484	**＜0.001**

AUC : area under curve; SUR: standardized uptake ratio; T/A : tumor/aorta.

**Table 3:** Comparison of the combination with conventional and texture parameters to only conventional parameters with IDI and NRI.

Variable	AUC	Sensitivity (%)	Specificity (%)	Youden index	*P*	IDI	95% CI	*P*	NRI	95% CI	*P*
C + T	0.810	85.7	77.8	0.635	**＜0.001**	0.236	0.095–0.377	**0.001**	0.434	0.030–0.838	**0.035**
Only C	0.788	71.4	77.8	0.625	**＜0.001**						

C + T : conventional metabolic parameters plus texture parameters; only C : only conventional metabolic parameters; AUC : area under the curve; IDI : integrated discrimination improvement; NRI : net reclassification improvement (categorical); 95% CI: 95% confidence interval.

## Data Availability

The data used to support the findings of this study are available from Guanyun Wang (E-mail: 852791126@qq.com) upon reasonable request.
